# Risk factors for poor health and performance in European broiler production systems

**DOI:** 10.1186/s12917-020-02484-3

**Published:** 2020-08-12

**Authors:** Tommy Van Limbergen, Steven Sarrazin, Ilias Chantziaras, Jeroen Dewulf, Richard Ducatelle, Ilias Kyriazakis, Paul McMullin, Jesús Méndez, Jarkko K. Niemi, Sotiris Papasolomontos, Piotr Szeleszczuk, Johan Van Erum, Dominiek Maes, on behalf of the PROHEALTH consortium

**Affiliations:** 1 Department of Reproduction, Obstetrics and Herd Health, Faculty of Veterinary Medicine, Ghent University, Salisburylaan 133, 9820 Merelbeke, Belgium; 2 PEHESTAT BVBA, Dwarsstraat 3, 3560 Lummen, Belgium; 3 Department of Pathology, Bacteriology and Poultry Diseases, Faculty of Veterinary Medicine, Ghent University, Salisburylaan 133, 9820 Merelbeke, Belgium; 4 Agriculture, School of Natural and Environmental Sciences, Newcastle University, Newcastle upon Tyne, NE1 7RU UK; 5 Poultry Health Services International, 4 Cocked Hat Park, Sowerby, Thirsk, North Yorkshire YO7 3HB United Kingdom; 6 Cooperativas Orensanas S.C.G, Santa Cruz de Arrabaldo, s/n, 32990 Ourense, Spain; 7 Natural Resources Institute Finland (Luke), Kampusranta 9, FI-60320 Seinäjoki, Finland; 8 Vitatrace Nutrition Ltd., Propylaion 18, Strovolos Industrial Estate, 2033 Nicosia, Cyprus; 9 Department of Pathology and Veterinary Diagnostics, Division of Avian Diseases, Warsaw University of Life Sciences (SGGW), Nowoursynowska 166, 02-787 Warszawa, Poland; 10 Galluvet bvba, Dwarsstraat 3, 3560 Lummen, Belgium

**Keywords:** Broiler health, European broiler production, Housing, Risk-factors, Statistical modelling

## Abstract

**Background:**

Conventional broilers are currently one of the most efficient protein converters. Although decades of progress in genetic selection and feed formulation have lead to high standards of efficient broiler production, still a lot of variability is found between farms and between successive flocks. The aim of this study was to investigate risk- and/or protective factors for poor health and performance in conventional broiler-farms in Europe by developing eight multivariable linear mixed models. Three different models were used to investigate mortality (overall, first week, after first week), three models for performance variables (growth, feed conversion, European production index) and two models were related to slaughterhouse data (i.e. dead on arrival and condemnation rate).

**Results:**

Several factors related to management and housing were significantly associated with health and performance of broilers. The following factors were associated with increased mortality: floor quality, neonatal septicemia, ventilation type and other professional activities of the farmer. The factors associated with performance were chick sex, coccidiosis infections, necrotic enteritis, dysbacteriosis, light intensity adaptations, ventilation type, comparing daily flock results with previous flock results by farmer, daily check of feed and water system and type of feed. For dead on arrival three risk factors were identified i.e. daily growth, type of light adaptation and type of drinkers system. For condemnation rate seven risk factors were found, i.e. type of drinking system, daily growth, feed withdrawal time, type of ventilation, house size, septicemia after seven days and type of feed.

**Conclusions:**

These results imply that a multifactorial approach is required with adaptations involving both improvements in management, housing, health programs and an increasing level of professionalism of the farmer in order to improve broiler performance and health.

## Background

Poultry meat production has grown rapidly during the last 40 years and is expected to continue to grow [1]. The demand for meat is increasing because of population growth, rising income and urbanization, and poultry meat has shown the fastest rise in the last decades. In order to be as sustainable as possible, the ecological footprint of broiler meat production needs to be restricted. In comparison to broiler production in the sixties, current broiler production already requires fewer resources: fossil energy use (− 39%), water use (− 58%), and agricultural land occupation (− 72%) per 1000 kg of poultry meat produced [2]. The industry has been very successful in improving bird performance and sustainability, and further improvements continue to be implemented. However,the gap between the potential of the birds and the actual performance achieved under practical conditions is the field is getting wider. A large number of infectious and non-infectious risk factors have already been identified to cause decreased performance and increased mortality in conventional broilers [3].

Avian pathogenic *E.coli* (APEC) for example, has been known for decades to cause disease and mortality in broilers, APEC is also associated with high rates of antibiotic resistance [4, 5]. APEC can persist in a dry environment, and dust in poultry houses may contain up to 10^6^ colony-forming units of *E. coli* per gram [4, 6]. The most important predisposing factor for APEC infections in broilers however is stress, which can be induced by a series of inappropriate husbandry and management practices [7]. However, there are only few epidemiological studies taking a more integrated approach with multivariable modeling in order to identify and weigh the different risk factors threatening performance and health of broilers in field conditions without focusing on only one or a few specific preselected diseases or clinical features. Flock size, stocking density, use of paper underlay for feeding during the 1st week for example have been identified as risk or protective factors for first week mortality [8, 9]. The same study also identified that age of the broiler house, heating system and control system for the air intake could be associated with broiler mortality after seven days. A study from Taiwan investigated risk factors for first week mortality and identified other risk and protective factors such as water cooled ventilation, high flock size and distance from hatchery < 50 km [10].

Unfortunately, many experimental studies investigating the factors that can influence broiler production have not taken into account the dynamic nature of these processes, by only focusing on specific measurements taken at a specific point in time (e.g. investigating footpad dermatitis at slaughter), often leading to conflicting results [11–14]. Therefore there is a strong need for studies targeting the multivariable aspects of the European broiler production using high quality data, covering the main conventional broiler producing countries of Europe, and taking into account specific random and fixed factors (e.g. seasonal influences and country specific production systems) in order to find robust risk factors for conventional broiler production systems throughout Europe.

The main novelty of this current study is that it investigates and quantifies risk- and/or protective factors for different health and performance parameters in conventional broiler-farms in multiple EU member states using a standardized way of data-collection and analysis.

## Results

### Descriptive results

#### General farm characteristics

A total of 358 broiler-farms were included in this study. Seventy-eight percent (*n* = 278) of the farms stated that the production of broilers was their only activity on the farm; 80.1% (*n* = 281) was part of a quality assurance scheme and 52.8% (*n* = 186) were part of an integrated system with some degree of standardized management procedures.

#### Housing

An overview of the descriptive results for subcategories “Floor quality”, “Ventilation” and “Heating” is provided in Table 1. The average broiler farm contained three broiler houses of 24 years old with each of them housing approx. 24,000 broilers using an average density of 17.5 day old chicks per square meter. Almost half (*n* = 160) of the mechanically ventilated houses also contained specific ventilators meant for the recirculation of air inside the broiler house. Natural ventilation was still present in 7.3% (*n* = 26) of broiler farms. A cooling system of some type was present in 71.5% (*n* = 256) of all broiler farms for the prevention of heat stress in broilers. The most common type of cooling system was based on the creation of a fog in the neighbourhood of the air inlets of the broiler house, i.e. 78.8% (*n* = 201), of which the position of the fog system was located inside the house in 79.6% (*n* = 160) of the cases. Pad cooling was present in 20.7% (*n* = 53) of farms that had a cooling system.

**Table 1:** Descriptive results of the housing of the broiler farms included in the study

Subcategory	Situation on farm	Number of farms^d^	% of total
Floor quality^a^	Smooth impervious	132	37.7
	Fair condition	46	13.1
	Cracked to some degree	165	45.9
	Compacted earth	15	4.2
Ventilation^b^	Roof ventilation	104	29.1
	Cross ventilation	74	20.7
	Roof x Tunnel ventilation	72	20.2
	Tunnel	40	11.2
	Cross x Tunnel ventilation	37	10.3
	Natural ventilation	26	7.3
	Other	5	1.4
Heating system^c^	Direct	194	54.3
	Indirect	164	45.7

^a^Floor quality was scored into four categories from “smooth impervious” (best situation) to “compacted earth” (worst situation). ^b^The type of ventilation system used to refresh the air inside the broiler house. ^c^The heating system was classified into two categories: direct heating (CO_2_ is produced inside the broiler house) and indirect heating (no CO_2_ is produced inside the broiler house. ^d^ Number of farms that have this situation on farm, a total of 358 broiler farms participated in this study

#### Feed and water supply

Two types of feed systems were used in broiler farms: pan feeders (*n* = 268, i.e.74.9%) and feeding troughs (*n* = 90, i.e.25.1%). Pan feeders had an average length of 1.97 cm per broiler and with feeding troughs an average length of 2.74 cm was available per broiler (based upon the number of broilers placed in the broiler house). The type of feed provided to broilers was a mixed feed compound in 199 farms (i.e.55.6%), while the other farms used a concentrated feed and added other ingredient (e.g., whole wheat (150 farms, i.e. 94.3%), maize (2 farms, i.e. 1.3%) or a combination of whole wheat and maize (7 farm, i.e.4.4%)). The main sources of drinking water were municipal water (*n* = 158 farms, i.e.44.0%) and by using a ground well (*n* = 188 farms, i.e. 52.4%), for ground wells the average depth was 91.9 m. About half of the farms (*n* = 177, i.e. 49.4%) used water disinfection protocols to sanitize the drinking water, the most commonly used chemicals for disinfection were chlorine dioxide (*n* = 90, i.e. 51.1%) and peroxides (*n* = 39, i.e. 22.2%). The drinking system of the investigated broiler farms was on average 16 years old. Over 89% of all broiler farms (i.e. 319 farms) used nipple drinkers with 13.3 chicks per drinking nipple. A minority of the farms was equipped with cup drinkers (*n* = 20, i.e. 5.6%) or round drinkers (*n* = 19 farms, i.e. 4.8%).

#### Treatments and diseases

An overview of the responses regarding the main health problems in the investigated broiler farms is provided in Table 2. The majority of investigated broiler farms used a standard anti-coccidial treatment in the feed (346 farms, i.e. 96.6%). Vaccination against coccidiosis was performed in all flocks in only 6 farms (i.e. 1.7% of farms). Alternating vaccinated against coccidiosis and flocks treated with anti-coccidials was done in 42 farms (11.8% of broiler farms). The most frequently used anti-coccidials in starter feed were narasin-nicarbazine (*n* = 197, 54.9%), nicarbazine (*n* = 58, 16.2%), monensin-sodium (*n* = 39, 11.0%), narasin (*n* = 26, 7.2%) and salinomycin (*n* = 23, 6.3%). For grower feed the most commonly used anti-coccidials were monensin-sodium (*n* = 151, 42.1%), narasin-nicarbazine (*n* = 121, 33.7%), salinomycin (*n* = 38, 10.7%) and narasin (*n* = 31, 8.6%). In finisher feed the main anti-coccidials were narasin (*n* = 219, 61.1%), monensin-sodium (*n* = 55, 15.5%), salinomycin (*n* = 43, 12.0%) and narasin-nicarbazine (*n* = 24, 6.6%). When a withdrawal period was required for the anti-coccidial, the feed was on average two days free of anti-coccidial before loading (thinning included). Thinning of the flock before complete emptying of the broiler house was done in 70.1% of all broiler farms (*n* = 251). About half of these broiler farms (*n* = 125, 49.8%) restarted with in feed-anti-coccidials after thinning.

**Table 2:** Number and % of farms with health problems in 2016

Health problems	No problem		Mild problem		Clinical problem	
	N^a^	%	N^a^	%	N^a^	%
Coccidiosis	232	64.9	94	26.3	32	8.8
Septicemia before 7 days of age	149	41.6	57	15.9	152	42.4
Septicemia after 7 days of age	154	43.0	61	17.0	143	39.9
Dysbacteriosis	217	60.6	94	26.2	47	13.1
Necrotic enteritis	274	76.4	68	18.9	17	4.7
Wet litter syndrome	145	40.4	190	53.1	23	6.5
High mortality	284	79.2	56	15.7	18	5.1
Bad flock uniformity	189	52.7	160	44.7	9	2.6

^a^Number of farms, in total 358 farms participated in the study

#### Specific broiler information

Approximately 56.9% of all questioned broiler farmers (*n* = 204) knew the age of the breeder parent stock from which their day old chicks originated. The initial bodyweight of day old chicks was known by 83.1% (*n* = 297) of all broiler farmers and 95% (*n* = 282) of them used such information to adapt the production management e.g. by using a higher set temperature in case of a low initial bodyweight. The transport time between the departure of day old chicks from the hatchery and the arrival in the broiler house was less than 4 hrs in 81.6% of all broiler farms (*n* = 292), while 2.8% of all farmers (*n* = 10) received day old chicks after more than 8 hrs. The majority of broiler farms (*n* = 255) always received day old chicks as hatched (71.3%), i.e. with no sex differentiation.

#### Production management and performances

An overview of different aspects of the production management of the farms included in the study was provided in Table 3. Farmers tended to start preheating their broiler houses on average 34.7 h before placement with an average set temperature of 33.2 °C. During the first three days after placement, a lighting schedule of an average of 68.2 h of light (i.e. 94.2% of the time) was used in the broiler house. During the rest of the production period, an average of 5.9 h of darkness per 24 h was provided to the broilers.

**Table 3:** Descriptive results of the production management of the broiler farms included in the study

Subcategory	Situation on farm	Number of farms^e^	% of total
Date of placement^a^	Complete area of the house^c^	206	57.5
	Floor temperature measured^d^	263	73.6
Light intensity	Adapted during production cycle	245	68.5
Litter material	wood shavings	102	28.5
	cut straw	90	25.1
	peat	46	12.8
	rice hulls	39	10.9
	complete straw	31	8.7
Daily data registration	water intake	314	87.7
	feed intake	146	40.8
	bodyweight	97	27.1
Light during catching^b^	Adaptation of light	349	97.5
	- Decrease of light intensity	181	51.8
	- Use of red lights	105	30.1
	- Use of blue lights	63	18.1

^a^Date when day old chicks are placed into the broiler house; ^b^Light that is used when broilers are caught for transport to slaughterhouse; ^c^Day old chicks have access to the full floor area of the broiler house; ^d^Floor temperature is measured when day old chicks are placed into the house; ^e^Number of farms that have this situation on farm

At 15.5% of broiler farms (*n* = 55) extra drinkers were provided to the day old chicks and these were removed after 4.9 days on average. Almost all broiler farms (*n* = 339, 94.6%) provided extra feeders for day old chicks. The majority of broiler farmers (*n* = 273, 76.3%) checked crop fill of chicks during the first 24 h after arrival on the farm. The average feed withdrawal time before loading was 7.3 h, while water was withdrawn only very shortly before loading, i.e. on average 0.5 h before loading. A number of parameters can be registered automatically on a daily basis in broiler farms, farmers were questioned about the registration of average body weight, average feed intake and average water intake (see Table 3). The majority of farmers (*n* = 348, 97.2%) compared the recorded information with data from previous batches or general schemes. The flow rate of the drinking system was checked daily by 53.9% of all farmers (*n* = 193), while 42.2% of farmers (*n* = 151) did not check water flow rate every day but checked flow rates as soon as abnormal fluctuations in the daily water intake were observed. Drinking nipples and feeding system were checked daily by 94.1% of the farmers (*n* = 337). Almost all broiler farmers (*n* = 352, 98.3%) stated that when abnormal birds (e.g. runt or lame birds) were observed during daily inspection, these animals are culled. Forty percent of the questioned farmers (*n* = 143) stated that they received no information at all from the slaughterhouse regarding health and/or welfare parameters of their broilers.

A summary of the specific health and performance parameters for broiler farms is provided in Table 4. These values are based on 2309 flocks from 358 broiler farms.

**Table 4:** Performance and health parameters based on 2309 flocks from 358 broiler farms in 2016 originating from 7 EU member states

Parameter	Average	Median	SD^a^	Minimum	Maximum
Average age at slaughter (days)	41.33	41	3.62	30.26	58.01
Average weight at slaughter (kg)	2.47	2.50	0.43	1.66	3.31
Overall mortality (%)	3.82	3.70	1.40	1.00	14.86
First week mortality (%)	0.94	0.90	0.51	0.03	3.29
Dead on arrival (%)	0.20	0.14	0.37	0.01	4.60
Condemnation rate (%)	1.23	1.00	0.92	0.05	6.73
Feed conversion rate	1.74	1.70	0.17	1.23	2.06
Daily gain (gram/day)	59.79	60.62	5.89	41.19	72.95
EPI^b^	338.41	345.85	53.07	183.93	432.17

^a^Standard deviation; ^b^European Production Index, calculated by multiplying average bodyweight with livability, dividing this result by the product of FCR and average age. This result multiplied with “100”, provides the EPI. Livability is defined as the percentage of the total number of broilers at placement that reaches slaughter-age

### Risk factor analysis

**Fig. 1:**
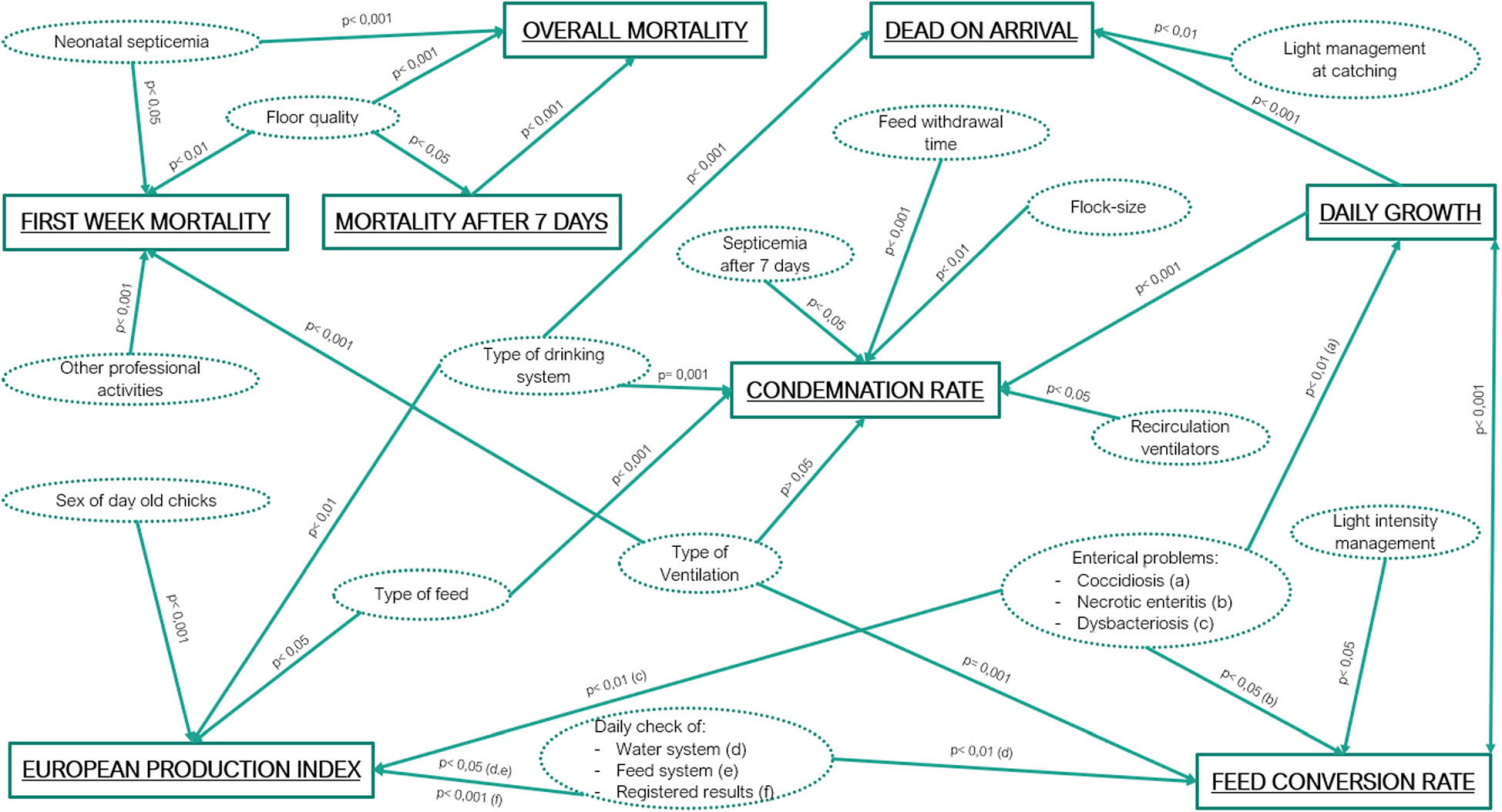
Causal pathway with statistical significant associations in the multivariable models between several management, performance, housing and health variables in broiler farms. Full lines represent the result of a multivariable linear regression analysis based on data from 7 EU countries. The *p*-values correspond to the multivariable model. All models were corrected for the country effect by placing country as a fixed variable in the model

Eight linear mixed models were fitted based on these health and performance parameters. A summary of the significant risk factors is provided in Table 5, an overview of the relation between all significant factors is provided in Fig. 1.

**Table 5:** Multivariable linear mixed models related to performance and health parameters in 2309 flocks from 358 broiler farms in 7 EU member states

Model	Dependent variable	Independent variable^a^	*p*-value	*B*-value
**1**	**Overall mortality rate**	Mortality rate after 7 days	< 0.001	1.03
		Floor quality^b^	< 0.001	- 0.72
		Neonatal septicemia	< 0.001	0.22
**2**	**First week mortality rate**	Floor quality	0.004	- 0.67
		Ventilation type^c^	< 0.001	0.72
		No other professional activities by farmer	< 0.001	0.24
		Neonatal septicemia^d^	0.014	- 0.12
**3**	**Mortality rate after seven days**	Floor quality	0.046	- 0.35
**4**	**Dead on arrival**	Light management during catching^e^	0.003	0.09
		Type of drinking system	< 0.001	- 0.41
		Daily growth (g/d)	< 0.001	0.01
**5**	**Condemnation rate**	Type of drinking system	0.001	- 1.40
		Daily growth (g/d)	< 0.001	0.05
		Feed withdrawal time	< 0.001	- 0.12
		Number of birds in the broilerhouse	0.002	0.02
		Presence of recirculation vents	0.039	0.25
		Septicemia after seven days	0.012	0.23
		Type of feed	< 0.001	0.75
		Ventilation type^c,f^	0.057	- 0.31
**6**	**Feed conversion rate**	Daily growth (g/d)	< 0.001	- 0.01
		Light intensity adaptations	0.013	0.02
		Ventilation type^c^	0.001	- 0.03
		Necrotic enteritis problems	0.014	- 0.02
		Daily check of drink water flow	0.002	- 0.03
**7**	**Daily growth**	Feed conversion rate	< 0.001	- 29.35
		Coccidiosis problems	0.004	1.33
**8**	**European production index**	Sex of day old chicks^g^	< 0.001	34.54
		Dysbacteriosis problems	0.002	14.97
		Evaluation of daily registered results	< 0.001	- 33.94
		Daily inspection of feed and water system	0.029	- 12.80
		Type of drinking system^h^	0.006	54.37
		Type of feed^i^	0.024	- 13.01

^a^Only the statistically significant risk factors in the final models are presented; ^b^The reference used for floor quality was a floor in perfect conditions without cracks; ^c^The reference used for type of ventilation was roof ventilation; ^d^This was only the case in tunnel-ventilated broiler houses; ^e^The reference for light adaptation was dimming the light intensity; ^f^Interpreted as a trend, as it was not significant (*p* > 0.05); ^g^If only male chicks were housed, a higher EPI was present; ^h^The reference used for drinking system was the nipple drinking system; ^i^Only the case when dysbacteriosis was absent in the flock

The first linear model included overall mortality as the dependent variable. The following three independent variables were identified as potential risk factors: mortality rate after 7 days; floor quality and the occurrence of neonatal septicemia on the farm. The higher the mortality rate after 7 days, the higher the overall mortality was in the investigated flocks. Poor floor quality was a significant risk factor for overall mortality in broilers as farms with lower quality floors with cracks, in their broiler houses appeared to have significantly higher overall mortality rates. The third risk factor for overall mortality was the occurrence of problems with neonatal septicemia.

Four significant risk factors were found for first week mortality: floor quality, ventilation type, presence of other professional activities of the farmer and the occurrence of problems with neonatal septicemia. The risk was higher in farms with cracked floors compared to floors without cracks, in farms using tunnel or roof ventilation, in farms in which the farmer had multiple professional activities and in farms experiencing neonatal septicemia in combination with tunnel ventilated broiler houses.

For mortality after 7 days, only one significant risk factor was found i.e. poor floor quality (cracks are present in the floor in which pathogens might survive from previous flocks) of the.

The analysis for farm specific risk factors for high number of dead on arrival (DOA) to slaughterhouse identified three significant variables: the type of light adaptation when broilers are caught, the drinking system and the daily growth during the cycle. The use of red lights for catching broilers increased the risk for dead on arrival in the slaughterhouse in comparison with decreased light intensity. The other methods of light adaptation had no significant effect. Other significant risk factors were not using nipple drinkers system and higher daily growth.

Seven significant risk factors and one borderline non-significant factor could be associated with condemnation rate: drinking system, daily growth, feed withdrawal time, ventilation system, average house size, the presence of recirculation vents, problems with septicemia after the first seven days and the type of feed that was used during the grower phase. The usage of cup drinkers was associated with a lower condemnation rate in comparison with nipple drinking systems, while no significant effects were noticed for the other types of drinkers. A significant positive association was found between increasing daily growth and increasing condemnation rate at slaughterhouse. Sufficient feed withdrawal time was associated with lower condemnation rates. Condemnation rates further increased with higher capacity of a broiler house (i.e. the number of birds that can be housed in the same airspace), in case no recirculator vents (which are used to mix up the air in a broiler house) were present in the broiler house, when there were septicemia problems after seven days of age and in case of concentrate + wheat was used compared to the use of complete compound feed. Although not statistically significant, there was a trend that condemnation rate was lower in cross ventilated broiler houses in compare to roof ventilated houses.

Feed conversion rate (FCR) was significantly associated with five variables: daily growth (DG), the possibility to change light intensity, type of ventilation, the occurrence of necrotic enteritis (NE) and daily check of drinking water flow rate. A clear significant negative association was found between DG and FCR. Feed conversion rate was significantly higher in broiler houses that could not adapt the light intensity of the broiler house. A clear effect of the type of ventilation system was found on FCR. Roof ventilated broiler houses appeared to have the best FCR, followed by cross ventilated and cross x tunnel ventilated broiler houses. FCR was significantly lower in mechanically ventilated broiler houses compared to natural ventilated broiler houses. The absence of problems associated with NE was associated with a significantly better FCR. FCR was significantly better in farms that did not perform a daily check of the flow rate of the drinking system, but systematically checked the flow rate of the drinking system in case abnormal fluctuation of the water intake occurred.

For DG only FCR and the occurrence of problems with coccidiosis infections were risk factors. A negative association was found between FCR and DG. Farms that stated to have no problems with coccidiosis during the production period were found to have a significantly higher DG.

Six variables significantly influenced European Production Index (EPI): Sex of day old chicks (male chicks had a higher EPI), the occurrence of problems with dysbacteriosis, if farmers compared their daily registered results with results from previous batches, if farmers checked the drinkers and feeders on a daily basis, the type of drinkers and the type of feed. The EPI was significantly higher in broiler farms that only housed male broilers compared to farms that housed only female broiler or a mix of both genders (as hatched). Farms that only housed female broilers had a significantly lower EPI. Farms that encountered no problems with dysbacteriosis had a significantly higher EPI compared to farms that had problems with dysbacteriosis. Farmers that did not compare their recorded information (e.g. bodyweight, feed- and water intake) with data from previous batches or general schemes had a significant lower EPI in comparison with farmers that compared flock results. Farmers who stated that they did not check the feeders and drinkers frequently had a significant lower EPI compared to farmers who did this on a daily basis. Farms with nipple or cup drinkers had a better EPI compared to farms with round drinkers. There was no difference between farms with nipple and cup drinkers. Broiler farms that had no problems of dysbacteriosis and used a concentrate with whole wheat, had a significant lower EPI compared to farms without problems of dysbacteriosis that used a complete compound.

## Discussion

This study identified different management, housing and health factors as risk or protective factors for broiler performance and health on conventional broiler farms in seven EU member states.

Full randomization in the selection of the farms was not always possible, a selection-bias is therefore present to some extent. The mode of questionnaire administration was identified before to have clear effects on data-quality [15]. Data collection by farm visits was encouraged, allowing a personal contact between the interviewer and the poultry farmer. Although farm visits were set as the standard method for data collection some data from two out of seven countries were collected via conventional mail or telephone interviews. A potential effect on data-quality by these different modes of data collection in these two countries could therefore not be excluded. The researchers involved in the data collection in both countries have cross-checked the replies to the questionnaires, when possible, with previously recorded data, to limit the potential loss of data-quality. This questionnaire was developed for this study in order to collect details about farm housing and management practices in a standardized way in all participating farms. The possible interviewer bias was limited as much as possible by limiting the number of people responsible for the collection of data and also by training the interviewers to follow a specific predesigned procedure during the farm visit. The relatively high number of farms that was included in this study increases the representativeness of the study population. The farm characteristics in the seven participating member states were in accordance with previous publications [16, 17].

Three different models were constructed to investigate mortality rate in broilers, i.e. overall mortality, first week mortality and mortality after seven days. This classification was used because previous research about mortality rate in average broiler production was shown to follow typical patterns with a peak in mortality at day three and four after placement, a stable low mortality rate during the rest of the production cycle with a slight increased mortality rate during the sixth week of broiler production [18–21]. For this reason a distinction was made between mortality rate before and after seven days of placement [8]. It was impossible to distinguish the effect of culling in the current dataset. Culling programs might vary between different broiler producers and culling is used to remove birds that are about to succumb [19, 22]. A proper culling program has beneficial effects on the FCR and flock uniformity [22].

In the current study, all types of mortality were associated with poor floor quality. Poor floor quality included the presence of cracks in the floor. The floor of poultry houses is mainly made of concrete. Cracks in the concrete floor of a poultry house create an environment which is impossible to be properly cleaned and disinfected between successive flocks and which can allow multiple pathogens (e.g. *E.coli* or *Eimeria spp*.) to spread and cause disease in successive flocks [23]. At the same time cracks can also house bacteria which are resistant to antimicrobials, causing reduced therapeutic effects in case an antimicrobial treatment is required, thus causing elevated mortality [20, 24].

Neonatal septicaemia was positively associated with both a higher overall and first week mortality. Avian pathogenic *E.coli* (APEC) has been known for decades to cause disease and mortality in broilers and is the main cause for neonatal septicemia resulting in elevated mortality rates [4]. APEC can persist in a dry environment, and dust in poultry houses may contain up to 10^6^ colony-forming units of *E. coli* per gram [6]. The most important predisposing factor for APEC infections in broilers is stress, which can be induced by a series of inappropriate husbandry practices [7].

First week mortality was also positively associated with the use of tunnel or roof ventilation in broiler houses. The use of tunnel ventilation appeared to have a higher association with first week mortality compared to the use of roof ventilation. This result is confirmed by two other studies, who found a higher first week mortality in broiler houses with negative pressure ventilation compared to naturally ventilated houses [8, 10]. However, a more recent Spanish study found no influence of ventilation on first week mortality [9]. These three studies are all studies conducted within one country (Norway, Taiwan and Spain resp.), possibly with less variation in climatic and/or housing conditions between the farms within the country. The climate can largely influence the impact of ventilation type on first week mortality. The present study included the effect of climate, and farms located in countries with different climates (moderate, Mediterranean, Scandinavian) were represented.

When the farmer had other professional activities besides broiler production, the first week mortality appeared to be higher compared to farmers for whom broiler production was their sole professional activity. A possible explanation for this finding is that the farmers had less time available for the preparation of a broiler house and the management during the first week after placement. The pre-placement preparation of the broiler house and the first days after placement might require crucial interventions by the farmer based upon the birds’ needs, e.g. adaptations of the house temperature [8, 10, 20, 21]. The combination of broiler production with other professional might therefore lead to a less strict follow up of broilers, which might lead to a higher first week mortality.

Although several risk factors were identified to be related with the different types of mortality, previous studies identified additional risk factors. A Norwegian study by Heier et al. [8] identified flock size, stocking density, use of paper underlay for feeding during the first week as risk- or protective factors for first week mortality. The same study also identified age of the broiler house, heating system and control system for the air intake as risk factors for broiler mortality after seven days. A study from Taiwan investigated risk factors for first week mortality and identified other risk and protective factors such as water cooled ventilation, high flock size and distance from hatchery < 50 km [10]. The difference between the current study and the other studies that were mentioned is due to different regional and climatic conditions in these studies and the inclusion of multiple climatic conditions in the present study.

The model that was designed to investigate risk factors for DOA identified three significant risk or protective factors. The use of red lights during catching appeared to result in a higher number of DOA compared to other types of light management, i.e. dimmed lights or blue lights. This was in accordance with the results of Prayitno et al. [25], who investigated the effects of color of lighting, i.e. blue, green, red or white light, on the behaviour and production of broilers. Prayitno et al. [25] found that broilers were more active in red light and showed greater floor-pecking, wing-stretching and aggression in the red light when compared to blue or green light. Birds kept in green or blue light were calmer, which is beneficial to reduce stress when broilers are caught. The physiological explanation for the increased activity in broilers exposed to red light is that the hypothalamus is more stimulated in comparison to broilers exposed to blue or green light, resulting in an enhanced “flight or fight” response. The use of nipple drinkers was found to be associated with a less DOA. However, this type of drinkers system was present in almost 90% of the broiler houses and has become the new standard in broiler houses. Houldcroft et al. [26] indicated that when choice is offered, broilers tend to prefer bell drinkers or troughs over nipple drinkers in order to express the stereotypic “scoop” action while obtaining water. Nipple drinkers have the important advantage that less water is spilled. Wet litter can cause hock burn and pododermatitis [26]. An important aspect in nipple drinkers is the height of the drinking lines, which can be adapted according to the size of the broilers and was shown to have a significant impact on broiler performance [26–28]. Unfortunately, the latter could not be investigated in the current study design. It is important to state that the conditions during transportation and in the waiting area of the slaughterhouses were not included in this analysis although they may have an impact on the dependent variable: “dead on arrival”.

A high DG was associated with higher DOA, which is in accordance with previous studies, the main reason for this finding is that rapid growth rates can create oxygen deficits in broilers, leading to hypertrophy of the right heart ventricle, making these birds more susceptible for right-sided congestive heart failure and increasing the risk that catching and transport of these birds results in elevated mortality [29, 30]. Other studies have clearly identified transport of broilers from farm to slaughterhouse as a major risk factor for DOA [31–33]. In the current study, however, transport details after loading the broilers were not collected.

Condemnation rate in this study included all reasons for condemnation of carcasses in the slaughterhouse. It was not possible to obtain sufficient data from slaughterhouse reports which allowed a more detailed approach in investigating the different reasons for condemnation of broiler carcasses. Nevertheless our findings can provide indirect indications for the reasons why carcasses were rejected. The presence of cup drinkers in the broiler house was linked to a higher condemnation rate compared to nipple drinking systems. Cup drinkers are known to result in more water spillage than nipple drinkers [26]. Wet litter is known to induce skin lesions in broilers at the plantar surface of the feet (“footpad dermatitis”), the caudal aspect of the intertarsal joint (“hockburn”) and over the sternum (“breast blisters”) [26, 34]. In a recent study, skin lesions were shown to be a major reason for condemnation of poultry carcasses in French slaughterhouses [35, 36]. Increasing DG was associated with higher condemnation rates in our current study. Production of heavy broilers indeed results in a higher condemnation rate compared to the production of standard or light types of broilers [36]. The most likely explanation for this finding is that fast growing broilers have a higher incidence of subclinical heart disease and develop clinical signs of chronic heart failure and ascites, which can result in higher condemnation rates in fast growing broilers [37]. Feed withdrawal time was negatively associated with condemnation rate in broilers. This might be due to contamination of carcasses by the content of crop and digestive tract that is still present when the withdrawal period is too short [38]. Withdrawal of feed for 8 to 12 h before slaughter has the lowest risk for carcass contamination and has minimal losses for carcass weight [38, 39]. Flock size was also previously identified as a risk factor for condemnation rate in broilers [36]. There is no clear explanation for this finding in literature. However, a possible reason for this finding could be that in general good flock uniformity is more difficult to maintain in larger flocks, due to a potential higher variation in accessibility to food and water supply, small differences in the climate of the broiler house and other reasons related to management and housing of these large broiler houses. Abnormal flock uniformity results in a higher condemnation rate [35, 40]. The use of recirculation ventilators was found to be negatively associated with condemnation rate. The main beneficial aspect of such ventilators is that the air quality in the broiler house is more uniform and that a higher level of oxygen is available at the height of the broilers [41]. There was a trend for a lower condemnation rate in cross ventilated houses. A possible explanation might be the slightly lower air velocity in this type of ventilation. High air velocity (125 m/min) has indeed been identified to have a negative effect condemnation rates due to the negative impact on respiratory health [42]. When septicemia occurred after 7 days of age, there was a significantly higher condemnation rate. APEC is the main cause of aerosacculitis, polyserositis, septicaemia and other mainly extraintestinal diseases in broilers, turkeys and other avian species [43]. APEC are found in the intestinal microflora of healthy birds and most of the diseases associated with them are secondary to environmental and host predisposing factors [43]. The use of a concentrated feed with whole wheat added to it appeared to be associated with a lower condemnation rate. Whole wheat enhances feed efficiency because of a better utilization of nutrients in commercial feeds [44]. The use of whole wheat leads to higher relative weights of gizzard and pancreas [45]. A direct relation between whole wheat feed and condemnation rate is unclear, but the positive effects on gizzard and pancreas might indirectly result in a lower condemnation rate by a lower presence of runts.

FCR was lower in farms that were able to adapt light intensity in the broiler house. It is known that light intensity can influence the activity level of growing broilers [46]. Farms that can adapt light intensity in the broiler house can use this technique in their management to stimulate or slow down the feed consumption of broilers in case this is required according to breed standards and thereby improve performance parameters such as FCR. Mechanical ventilation was also found to be associated with a better FCR compared to natural ventilation. This is most likely due to a better air quality in general in mechanically ventilated houses as the growing chick has a high oxygen requirement in order to sustain rapid growth and optimal feed efficiency. Mechanical ventilation systems also better allow to adapt the ventilation volume to the needs of the growing broilers according to breed standards [47]. FCR was significantly higher in flocks that had necrotic enteritis problems. The latter was also shown in literature before by multiple authors, necrotic enteritis can include chronic intestinal mucosal damage which leads to poor digestion and increased FCR [48–50]. In flocks where farmers checked the flow rate of the drinking system in case of abnormal fluctuations a tendency to a better FCR was seen. Maiorka et al. [51] showed that the lack of water produced the same effect as the lack of feed, both causing a higher number of villi per area with reduction in villus size, when compared with feed and water ad libitum treatments, thus leading to inefficient absorption of nutrients and increased FCR.

The model for DG in broilers clearly showed that the presence of coccidiosis problems was a risk factor. Coccidiosis has been shown by many authors to negatively influence performance, the most important *Eimeria spp.* mainly colonize small intestine and due to their life-cycle, they cause major damage of intestinal epithelial cells, leading to an impaired function of the intestine and reduced DG [52, 53].

As expected the sex of the day old chicks appeared to have a significant effect on the EPI. The EPI was significantly higher in flocks that contained only male birds, when compared to as hatched flocks. When only female birds were housed, the EPI was lower than compared with as hatched flocks. This sex-difference in performance was also shown by previous studies. Male broiler birds are approximately 20% heavier than female broilers of the same age when fed ad libitum [54, 55]. Male broilers have an higher expression of agouti-related protein in the hypothalamus, which is suggested to be good indicator for the growth potential of a bird due to its modulating role in the central melanocortin system [56]. Farms that had recurrent problems of dysbacteriosis were found to have lower EPI. This is mainly due to the negative impact of dysbacteriosis on DG and FCR, which has comparable pathophysiological effects as described above for the impact of chronical necrotic enteritis on FCR [53, 57]. Both parameters are included in the formula that is used to calculate EPI, and thereby have a direct impact on EPI. In case no problems of dysbacteriosis were present, a lower EPI was found in flocks fed a concentrate with whole wheat. As described earlier, whole wheat enhances feed efficiency and leads to higher relative weights of gizzard and pancreas. In some cases however, it might also limit feed intake of broiler birds compared to the use of complete compound feed [44, 45]. The use of round drinkers was found to negatively influence the EPI. Round drinkers are known to lead to more water spillage, possibly negatively influencing water quality, and also to have a much lower water pressure. These factors might lead to suboptimal production of broilers. A direct negative effect of round drinkers on FCR or mortality was not found in this study nor in a previous study that specifically compared different types of drinking systems in broilers [58]. In our study the EPI was also influenced by the farmer’s stockmanship, i.e. comparison of daily results with results of previous cycles and the daily check of the feed- and water system. It is well known that the level of expertise varies significantly between farmers in terms of complying with the genetic and nutritional guidelines of the broiler birds and this may directly impact production results [59, 60]. Furthermore, this is also directly influenced by the integrated approach of farm guidance e.g. by specialised poultry veterinarians and nutritionists [61, 62].

## Conclusions

This study showed that broiler health and performance are strongly influenced by many factors directly related to the broiler farm’s management and housing. Also the impact of health problems caused by septicemia, coccidiosis and dysbacteriosis was shown to have a major negative influence on broiler performance. These health problems are known to have a multifactorial origin in which the presence of the primary pathogen on its own does not necessarily causes clinical problems and the impact of the primary pathogen on broiler production becomes higher when circumstances are in their favor. In this study a number of risk and protective factors for broiler health and performance were identified. For housing this mainly related to: floor quality, which was strongly related to the mortality rates; ventilation, which had a clear impact on first week mortality, condemnation rate and FCR; light management at catching, which strongly influenced the DOA to slaughterhouse rate; and the possibility to adapt light intensity throughout the cycle, which had positive effects on performance by reducing FCR. Management practices such as the level of professionalism, feed withdrawal time and light adaptations during the productive period also had an impact on broiler performance, i.e. first week mortality, condemnation rate and FCR.

This study clearly identified risk and protective factors related to management and housing on broiler health and performance. This study implies that multiple factors can be addressed in improving health and performance in broiler production and thereby making it more sustainable. This involves adaptations of management, housing and health programs and increasing the level of professionalism of the farmer.

## Methods

### Farms

This observational study was performed in seven broiler producing EU member states: Belgium, Cyprus, Finland, Greece, Poland, Spain and the United Kingdom as a part of the EU-funded project PROHEALTH. In each EU member state a selection ofapprox. 50 conventional broiler farms were included. Farms were selected by the locally assigned investigators with the aim to provide a representative population sample. It was agreed that all participating farms in a member state needed to be as representative for the conventional broiler-industry of that specific member state as possible, preferably by using a randomized sample. Broiler farms that were producing in free-range or bio-label conditions were excluded from the study. Upon request of the participating poultry companies and as agreed by the PROHEALTH consortium, member state codes were used throughout the analysis and the results in order to fully guarantee confidentiality for the participating poultry companies. A written informed consent was obtained from all participants in this study.

### Collection of farm data

All data were collected between February and August 2016. In Belgium, Cyprus, Greece, Spain and the United Kingdom, data were collected during farm-visits, in Poland, data were collected through conventional mail and telephone interviews, and in Finland through on-line survey and telephone interviews and from company databases. A protocol for the interviewers was developed by the first author. To ensure inter-farm comparability and to reduce bias by interviewer as much as possible, all interviews were taken by one person in each country. The specific interviewers for each member state were instructed about the protocol of the interview during one of the PROHEALTH consortium workshops prior to the start of the farm-visits to standardize the approach in the different countries. The questionnaire is available as an additional file.

Two types of data were collected from each broiler farm, i.e. potential risk factors for poor health and production were collected using a questionnaire, and data on performance and health were collected from at least six subsequent flocks, i.e. at least over one year (including potential seasonal effects). The questionnaire was specifically designed for this study and contained a total of 67 questions, including both multiple choice and open-ended questions. The questions were classified into six categories, i.e. general information, housing, feed and water supply, treatments and diseases, broiler details and management. The questionnaire was tested on 10 broiler farms prior to its use in this project.

Performance and health parameters included “average age” (in days), “average weight” (in kg), “overall mortality” (expressed as percentage), “first week mortality” (expressed as percentage), “dead on arrival” to slaughterhouse (DOA, expressed as percentage), “condemnation rate” in slaughterhouse (expressed as percentage), “feed conversion rate” (FCR, calculated by dividing total amount of feed consumed by the birds (in kg), by total live weight of birds at slaughter (in kg)), “daily weight gain” (DG, calculated by dividing the average live weight at slaughter by the average age of the birds at slaughter in days) and European production index (EPI), calculated by multiplying average bodyweight with livability, dividing this result by the product of FCR and average age. This result multiplied with “100”, provides the EPI. Livability is defined as the percentage of the total number of broilers at placement that reaches slaughter-age. Mortality in broilers in the current investigation was the sum of the number of dead birds that were found during daily inspection and the number of birds that were culled. Health problems that had occurred in one or more flocks during the twelve months before questioning were recorded. They included: coccidiosis, septicemia, dysbacteriosis, necrotic enteritis, wet litter syndrome, high mortality and poor flock uniformity.

### Data and statistical analysis

Eight linear mixed models were developed. The following factors were used as dependent variables: overall mortality, first week mortality, mortality after seven days, DOA, condemnation rate, FCR, DG and EPI. If necessary, transformation of the dependent variables was considered to obtain a normally distributed outcome variable [63]. A random effect for country was included to correct for clustering of farms within a member state. Initially, univariable linear mixed regression models between the dependent variables and each predictor variable were examined. Furthermore, for continuous predictor variables, the assumption of linearity was examined by the Loess curves between each individual predictor variable and the dependent variables and by the scaled residuals of the univariable models. If necessary, transformation of the predictor variables was considered. Then, the independent variables were used to build a multivariable linear regression model by a manual stepwise forward model building procedure. The predictor variables ‘age’ and ‘stocking density’ were maintained in the model to correct for possible confounding. Variance inflation factors were examined to check for multicollinearity when adding predictors to the models and in case of multicollinearity, the biologically most relevant predictor was retained in the final model. Statistical significance during this step was assessed at *P* < 0.05. The estimates of the significant predictor variables are presented with their corresponding 95% confidence interval. Finally, all biologically plausible two-way interactions were tested and removed when non-significant (*P* > 0.05). To check whether the assumptions of normality and homogeneity of variance had been fulfilled, scaled residuals were examined. Only the results of the final models are shown. Statistical analysis were performed in SAS 9.4 (SAS Institute Inc., Cary, NC, USA).

## Supplementary information

**Additional file 1.** PROHEALTH WP1 specific questionnaire for BROILERS. The questionnaire contains a total of 67 questions, including both multiple choice and open-ended questions. The questions were classified into six categories, i.e. general information, housing, feed and water supply, treatments and diseases, broiler details and management.

## Abbreviations

APEC: Avian pathogenic *E.coli*
DG: Daily Growth
DOA: Dead On Arrival in slaughterhouse
EPI: European Production Index
FCR: Feed Conversion Rate
NE: Necrotic Enteritis
