# Supplementary material for: Risk factors for poor health and performance in European broiler production systems
Source: BMC Vet Res. 2020 Aug 12;16:287. doi: 10.1186/s12917-020-02484-3 (PMC7425143; doi:10.1186/s12917-020-02484-3)
Supplement: Supplementary file 1 — Additional file 1. PROHEALTH WP1 specific questionnaire for BROILERS. The questionnaire contains a total of 67 questions, including both multiple choice and open-ended questions. The questions were classified into six categories, i.e. general information, housing, feed and water supply, treatments and diseases, broiler details and management. [file 12917_2020_2484_MOESM1_ESM.pdf]

Specific questionnaire for broilers (to achieve D 1.3)

Version to be used by PROHEALTH WP1 poultry partners involved in the broiler-part

Contents

General information (1–4) 2

Housing (5-12) 3

Feed and Water supply / quality (13-21) 6

Treatments / Diseases (22-31) 8

Broiler details (32-37) 12

Production management (38-67) 13

General information (1–4)

1. What is your task on the farm (person completing this questionnaire)?
    - Flock veterinarian
    - Responsible for the daily care of the animals
    - Responsible for the general management of multiple farms (E.g. in vertical or horizontal integration)
    - Other: ……

2. Is the production of broilers the only professional (agricultural or non-agricultural) activity of the farmer ?
    - Yes
    - No

3. Are you part of a quality assurance scheme which includes independent external inspection? E.g.: Belplume (Belgium); IKB Kip (The Netherlands); QS (Germany); ACP (UK)
    - Yes
    - No

4. Is the farm part of an integrated system in which management procedures are standardized?
    - Yes
    - No

Housing (5-12)

5. Please specify the age of the building (years), the capacity (birds) and the inside surface (m²) per poultry house:

Poultry house number:	Age of building (years):	Capacity (birds):	Inner surface (m²):
1.			
2.			
3.			
4.			
5.			
6.			

6. Which of the following provides the best description of the floor of your poultry house:

   - Compacted earth (replaced as required)
   - Smooth impervious concrete (with no cracks or open expansion gaps to trap organic material)
   - Concrete in fair condition with moderate superficial cracking
   - Rough uneven concrete with many cracks
   - Other: ….

7. How often is the drinking system (water pipes and nipples / cups / round drinkers) replaced?

   - Never
   - Every ……………… years.
     - Done lastly ……………………….. years ago.

8. What type of ventilation is used in the poultry house(s) ?

   - Natural ventilation system

     - Specify the poultry house number(s):

   - Forced ventilation system:

     - **Roof ventilation**

       - Specify the poultry house number(s):

     - **Cross ventilation**

       - Specify the poultry house number(s):

     - **Tunnel (Length) ventilation**

       - Specify the poultry house number(s)

     - **Combined type of mechanic ventilation**:

       - Please specify:

         - Roof x Tunnel (Length) ventilation

           - In poultry house numbers:

         - Cross x Tunnel (Length) ventilation:

           - In poultry house numbers:

     - **Other**:

       - Specify the poultry house number(s):

9. Are recirculation ventilators present inside the poultry house to remix the air inside?

   - Yes, in all poultry houses

   - Yes, but not in all poultry houses:

     - Please specify the poultry house numbers which have this kind of ventilators inside:

   - No

10. Can the ventilation be adjusted according to varying weather conditions?
    - Yes
      - Manually
      - Automatically
    - No

11. Is there a cooling system present in each broiler house?
    - Yes, please specify the type:
        - Misting:
            - Fog system placed inside building
                - Specify the poultry house number(s):
            - Fog system placed outside building
                - Specify the poultry house number(s):
            - Both in – and outside Fog system
                - Specify the poultry house number(s):
        - Pad cooling
            - Specify the poultry house number(s):
    - No,
        - Specify the poultry house number(s):

12. How does the heating system work?
    - Indirect heating (no production of CO_2 and water inside the broiler house)
        - E.g. Hydronic or Hot water systems (heated water is circulated to the houses on the farm via water pipes, finned tubes, floor heating, …..
    - Direct heating (production of CO_2 and water inside the broiler house)
        - E.g. Conventional petrol, LPG, ….. heating system, with heaters inside the houses.

Feed and Water supply / quality (13-21)

13. What is the origin of the drinking water?
    - Ground well
        - Specify the depth of the well: ……….. m
    - Surface water (also including the storage of rainwater, if rainwater is stored)
    - Municipal water (public supply)
    - Other:

14. Are disinfectants added to the drinking water during the growing cycle ?
    - Yes, please specify the type:
        i. Chlorine dioxide
        ii. Acetic acid
        iii. Formic acid
        iv. Lactic acid
        v. Peroxides
        vi. Blend of organic acids
        vii. Other:
    - No

15. Which type of drinking system is used?
    Please indicate the type of drinking system + the number of birds per nipple /cup or round drinker.

    - High flow rate nipple-lines (>60ml/minute)
        - Specify the poultry house number(s):
        - Number of birds / nipple:

    - Low flow rate nipple-lines with drip cups
        - Specify the poultry house number(s):
        - Number of birds / nipple:

    - Low flow rate nipple-lines without drip cups
        - Specify the poultry house number(s):
        - Number of birds / nipple:

    - High flow rate cup system (>60ml/minute)
        - Specify the poultry house number(s):
        - Number of birds / drinking cup:

    - Low flow rate drinking cup system
        - Specify the poultry house number(s):
        - Number of birds / drinking cup:

    - Round drinkers
        - Specify the poultry house number(s):
        - Number of birds / round drinker:

    - Other: ………………………………..
        - Specify the poultry house number(s):
        - Number of birds / drinking unit:

16. What type of feeding system is used ?
    - Feeding trough with chain
    - Feeding trough with spiral
    - Pan feeders with spiral
    - Pan feeders with chain
    - Other: ……………………..

17. Are the broilers fed manually or automatically (*e.g.* by a house computer)?
    - Manually
    - Automatically

18. How many birds are present per feeding location?
    - …… birds / pan feeder (in case of pan feeders)
    - …… cm / bird (in case of feeding troughs)

19. What type of feed is used?

**TYPE**		STARTER	GROWER	FINISHER
Complete compound				
Concentrate + Wheat				
Concentrate + Maize				
Concentrate + Maize + Wheat				
Other: ………………………..				

20. What is the structure of the feed?

**STRUCTURE**		STARTER	GROWER	FINISHER
Mash				
Crumble				
Pellets				
Other: ………………………..				

21. Are any feed additives used?
    - Yes,
        - Please specify:
            - Organic acids
            - Probiotics
            - Prebiotics
            - Other: …..
    - No

Treatments / Diseases (22-31)

22. Is there a medication dosing system present for each house separately ?

- Yes,
  - Water reservoir (e.g. 1000 l) with circulation pump
    - In poultry house number(s):
  - Water reservoir (e.g. 1000 l) without circulation pump
    - In poultry house number(s):
  - Automatic water proportioner (e.g. dosatron®)
    - In poultry house number(s):
  - Other: ….
    - In poultry house number(s):

- No dosing system is present
  - In poultry house numbers:

23. Which of the following diseases were considered to be a problem on your farm during the last year (multiple answers are possible)?

Disease / pathogen	No problem	Yes, subclinical problem	Yes, clinical problem
Coccidiosis			
Infectious bronchitis (IB)			
Infectious laryngotracheitis (ILT)			
Infectious bursal disease (IBD / Gumboro)			
Newcastle disease virus (NDV)			
Enterococcus infections			
First week *E. coli* infections			
E.coli infections in broilers older than one week			
Dysbacteriosis			
Necrotic enteritis			
Other:			
Other:			

24. Which of the following conditions do you consider to be a problem at your farm?

		No problem		Yes, mild problem		Yes, severe problem
Wet litter						
High mortality						
Locomotion problems						
Respiratory problems						
Bad flock uniformity						
Condemnations due to:						
- Polyserositis						
- Ascites						
- Liver lesions						
- Breast meat quality						
- Runts						
- Hockburn						
- Other: …………………….						
Other: ………………………..						

25. What vaccinations are performed from hatching to slaughter?
(Please provide both ages in case of repeated vaccinations)

Kind of vaccine		Yes / No	Age(s) (days)	
Newcastle disease virus (NDV)				
Infectious bronchitis virus (IB)				
Infectious bursal disease (IBD / Gumboro)				
Marek's disease				
Coccidiosis				
Other:				

26. Which anti-coccidial strategy is used?
    - None
    - Vaccination at hatchery-level
    - Vaccination at farm-level (please specify age of birds and method):
        - Age: ………….. days
        - Method:
            - Spray on feed
            - Spray on chickens
            - Drinking water
            - Other: …………………
    - Standard anti-coccidial treatment in the feed
    - Standard anti-coccidial treatment in the water
    - Other: ……………………………………………………………

27. Do you use a shuttle program alternating anticoccidial drug with vaccination (during the last year)?
    - Yes
    - No

28. In case of standard anti-coccidial treatment in the feed, what kind of active substance(s) was (were) used in the last production cycle (during the last year):
Multiple answers are possible. In case of a shuttle program, please specify the coccidiostat per feeding period.

Active substance:		Feeding period (starter/ grower/ finisher)
Amprolium (Amprol®)		
Decoquinate (Deccox®)		
Diclazuril (0,5%) (Clinacox® - Coxiril®)		
Halofuginone		
Lasalocid A (Avatec®)		
Maduramycin (Cygro®)		
Monensin-sodium (Coxidin®)		
Monensin (Elancoban®)		
Narasin (Monteban®)		
Narasin-Nicarbazine (Maxiban®)		
Nicarbazine (Koffogran®)		
Robenidine (Cycostat®)		
Salinomycine (Kokcisan®)		
Salinomycine (Sacox®)		
Salinomycine (Salinomax®)		
Semduramicin (Aviax®)		
Sulfadiazinum – Trimethoprimum (Tucoprim®)		
Other:		

29. How often do you change the coccidiostat program (after how many production cycles)?
    - After every production cycle
    - After ……………….. production cycles
    - Never, the same program is always used (during the last year)

30. In case a withdrawal period is required for the coccidiostat, how many days is the feed free of the coccidiostat before loading (thinning included):
    - ….. days

- In case of thinning, do you restart with coccidiostat drugs after thinning ?
    - Yes
    - No

31. In case no withdrawal period is required for the coccidiostat, how many days before (if any) is the feed free of coccidiostat ?
    - …. days

Broiler details (32-37)

32. Are the day old chicks (DOC) in each house always originating from one breeder parent stock (during the last year)?
    - Yes, always (100% of the DOC per house originated from 1 breeder-flock)
    - Yes, sometimes
    - No (always a mix of different broiler- breeder flock origins)
    - I don't know

33. Do you know the vaccination program of the breeder parent stock?
    - Yes,
        - Please specify the diseases for which maternal protection is expected:
            - **NCD** (Newcastle Disease virus)
                a. Yes
                b. No
            - **IB** (Infectious Bronchitis virus)
                a. Yes
                b. No
            - **REO** (Reovirus)
                a. Yes
                b. No
            - **IBD** (Infectious Bursitis Disease virus)
                a. Yes
                b. No
            - ***E. coli***
                a. Yes
                b. No
            - Other: ……………
            - Other: ……………
    - No

34. Do you know the age of the breeder parent stock from which the broilers originate?
    - Yes,
        - Do you use this information to adapt your production management (e.g. higher set temperature in case of very young breeder parent stock) ?
            - Yes
            - No
    - No,
        - Do you weigh DOC upon arrival ?
            - Yes
            - No

35. Do you know the average bodyweight of the DOC (reported by the provider of DOC or by weighing them upon arrival)?
    - Yes,
        - Do you use this information to adapt your production management (e.g. a higher set temperature in case of a low average bodyweight)?
            - Yes
            - No
    - No

36. How much time (in hours) elapses between the departure of the day-old chicks at the hatchery and the arrival inside the broiler house?
    - Less than 4 hours
    - Between 4 and 8 hours
    - Between 8 and 12 hours
    - Between 12 and 24 hours
    - More than 24 hours

37. What is the sex of the day old chicks?
    - Male and female (as hatched)
    - Male
    - Female

Production management (38-67)

38. Do the day old chicks have immediate access to the complete area of the house after placement?
    - Yes
    - No

39. Is the floor temperature measured at the time DOC are placed in the house(s)?
    - Yes
    - No

40. How many hours before placement do you start preheating the poultry house(s)?
    - ……..hours

41. What is the set temperature (°C) of the broiler house at time of placement?
    - …… °C

42. What is the light schedule per day during the first 3 days after placement of the DOC in the house?

After placement (hours)		Total period of light (hours)		Total period of dark (hours)
0 to 24 hours (day 1)				
24 to 48 hours (day 2)				
48 to 72 hours (day 3)				

43. What is the main type of light schedule that is used during the rest of the production cycle?
    - None (periods of light and dark are the same as outside)
    - 18 hours of light (18L) and 4 hours of dark (4D) and 2 times 1hour of dark (2x1D)
    - 18L – 6 D
    - 8L – 4D – 8L – 4D
    - Other, please specify: ……………………..

44. Is it possible to dim the light (change the light intensity) in the broiler house(s)?
    - Yes, in poultry house numbers:
        - Is the light intensity adapted to the production management of the broilers (*e.g.* lowered during spray vaccination, etc.)?
            - Yes
            - No

    - No, in poultry house numbers:

45. Are high frequent fluorescent lamps present in the broiler house(s)?
    - Yes:
        - in poultry house numbers: ……………….
    - No:
        - in poultry house numbers: ………………..

46. Are extra drinking locations provided for the day old chicks?
    - Yes
        - How long are these extra drinking locations provided for the DOC?
            - …………… days
    - No

47. Are extra feed locations provided for the day old chicks (*e.g.* paper with feed on top of it)?
    - Yes
    - No

48. Do you check crop fill of DOC during the first 24 hours after arrival?
    - Yes
    - No

49. What type of litter material covers the floor upon arrival of the DOC?
    - Wood shavings
    - Straw (complete)
    - Straw (cut)
    - Pellets of straw
    - Crushed pellets of straw
    - Flax
    - Rice Hulls
    - Coconut Fibers
    - Sand
    - Peat
    - Other, please specify: ……………………………..

50. Is extra litter material added during the growing cycle?
    - Yes:
        - Please specify:
            - Wood shavings
            - Straw (complete)
            - Straw (cut)
            - Pellets of straw
            - Crushed pellets of straw
            - Flax
            - Rice Hulls
            - Other, please specify
    - No

51. Is the extra litter material present inside the broiler house (e.g. in plastic big bags) at the time of placement of chicks?
    - Yes
    - No

52. How many hours before loading are the broilers withdrawn from feed?
    - ……. Hours

53. How many hours before loading is water withdrawn?
    - ……. Hours

54. Is average bodyweight registered daily (i.e. by an automatic scale inside the broiler house)?
    - Yes
    - No

55. Is average feed intake registered daily?
    - Yes
    - No

56. Is daily water consumption registered?
    - Yes
    - No

57. How often do you check the flow rate of the drinking system?
    - Every day (during daily inspection)
    - When abnormal fluctuations in the water intake occur
    - When abnormalities are observed in the birds (abnormal behavior, rise in mortality, etc.)

58. How often are the drinking nipples and feeders checked whether they work properly?
    - Only when something seems wrong in the broiler house
    - Daily
    - Weekly
    - Less than weekly
    - Never

59. What is the procedure when runts (small birds), lame birds etc., are observed during inspection?
    - Nothing, they are kept in the broiler house
    - They are culled (euthanized)
    - Small birds are removed to a "recovery pen"

60. Is the person in charge of euthanasia of sick animals properly trained to carry out euthanasia of broilers?
    - Yes, on farm training
    - Yes, by the vet or a specific organization
    - No (no formal training)

61. Are all persons in charge of the broiler care properly trained to detect sick animals, heat or cold distress or behavioral abnormalities?
    - Yes, in the farm
    - Yes, by the vet or a specific organization
    - No

62. How are broilers being caught when emptying the building for slaughter?
    - By hand
    - By machine
    - Other: ……….

63. Is the light adapted during the process of catching the broilers?
    - Yes:
        - please specify the technique that is used:
            - The light is dimmed as much as possible
            - Blue lights are used (people can see, birds capture it as complete darkness)
            - The intensity of light is increased
            - Other: ……………………………………

    - No

64. Do you compare recorded information (e.g. bodyweight, feed and water intake, …) with data from previous batches or general schemes?
    - Yes
        - In case deviations are noticed, do you try to intervene (e.g. by adjusting the management, notifying your flock veterinarian, technician…)?
            - Yes, always
            - Yes, sometimes
            - No
    - No

65. Do you use data which you have recorded to improve (multiple answers are possible):
    - FCR
    - Growth
    - Broiler health
    - Curative treatments
    - Mortality rate
    - Economic performance
    - other aspects: ……

66. Which information is provided by the slaughterhouse regarding health and/or welfare in broilers (multiple answers are possible)?
    - None
    - Carcass quality
    - Flock uniformity
    - Footpad dermatitis / hockburn
    - Other: …………………..

67. Is an alarm system used in case of loss of electricity (E.g. ventilation, feed, drinking system)
    - Yes
    - No
